# Nivel de conocimiento en estudiantes de pregrado de odontología sobre prescripción de analgésicos, antiinflamatorios y antibióticos en odontopediatría

**DOI:** 10.21142/2523-2754-1104-2023-173

**Published:** 2023-12-28

**Authors:** Ana Patricia Requejo-Bustamante, Guido Alberto Perona-Miguel de Priego

**Affiliations:** 1 Postgrado de Odontopediatría, Universidad Católica Santo Toribio de Mogrovejo. Lambayeque, Perú. 46193583@usat.pe, anapatriciarequejobustamante@gmail.com, gperona@usat.edu.pe , guidoperona54@gmail.com Universidad Católica Santo Toribio de Mogrovejo Postgrado de Odontopediatría Universidad Católica Santo Toribio de Mogrovejo Lambayeque Peru 46193583@usat.pe anapatriciarequejobustamante@gmail.com gperona@usat.edu.pe guidoperona54@gmail.com

**Keywords:** conocimiento, antiinflamatorios, antibióticos, odontopediatría, knowledge, anti-inflammatories, antibiotics, pediatric dentistry

## Abstract

**Objetivo:**

Determinar el nivel de conocimientos en estudiantes de pregrado de odontología sobre prescripción de analgésicos, antiinflamatorios y antibióticos en odontopediatría.

**Metodología:**

Este estudio fue de tipo descriptivo, transversal y observacional. La población estuvo conformada por un total de 84 estudiantes. El instrumento fue un cuestionario validado por un juicio de expertos, el cual contenía 22 preguntas, divididas en 2 partes: 11 preguntas sobre AINE y 11 preguntas sobre antibióticos, para conocer si el nivel de conocimiento fue adecuado o inadecuado.

**Resultados:**

El nivel de conocimiento respecto del uso de analgésicos y antiinflamatorios en los estudiantes de pregrado del ciclo XII es insuficiente (90,5%), seguido por los estudiantes del ciclo X (82,9%) y, finalmente, los del ciclo VIII (82,1%), ambos también insuficientes. Con relación al conocimiento sobre los antibióticos en el ciclo VIII, fue del 85,7%; en el ciclo X, del 68,6%, y en el ciclo XII, del 61,9%, insuficiente en todos los casos**.**

**Conclusiones:**

Los estudiantes de pregrado evaluados presentaron un nivel de conocimiento mayoritariamente insuficiente sobre el uso de analgésicos y antiinflamatorios.

## Abstract (en)

**Objective:**

To determine the level of knowledge about the prescription of analgesics, anti-inflammatories and antibiotics in pediatric dentistry of undergraduate dental students.

**Methodology:**

This study was descriptive, transversal and observational. The sample included 84 students. The instrument was a questionnaire validated by expert judgment which contained 22 questions divided into 2 parts, 11 questions on NSAIDs and 11 questions on antibiotics, indicating whether the level of knowledge was adequate or inadequate.

**Results:**

The level of knowledge in the use of analgesics and anti-inflammatories in undergraduate students of cycle XII was insufficient (90.5%), followed by 82.9% in cycle X and finally 82.1% in cycle VIII also insufficient. In relation to antibiotics in cycle VIII there was 85.7% insufficient knowledge, in cycle X it was 68.6%, and finally in cycle XII 61.9%, also was insufficient.

**Conclusions:**

The undergraduate students presented mostly insufficient level of knowledge in the use of analgesics, anti-inflammatories and antibiotics.

## INTRODUCCIÓN

La farmacología estudia las acciones y propiedades de los fármacos, así como su interacción a nivel sistémico. En lo que respecta al fármaco, se entiende como aquella sustancia química que interactúa con un organismo vivo ^1^.

En la literatura se ha descrito que los problemas relacionados con medicamentos pueden ser evitables hasta en un 80%, lo que puede mejorar al momento de la elección terapéutica. El odontólogo, y aún más el odontólogo pediatra, está facultado para prescribir y usar racionalmente los fármacos en pacientes pediátricos ^2^. Actualmente, los fármacos más prescritos son los antibióticos, que se definen como una sustancia que inhibe o elimina el crecimiento bacteriano. Otro de los medicamentos más usados son los antiinflamatorios no esteroideos (AINE), uno de los grupos de medicamentos más prescritos y utilizados a nivel mundial debido a sus propiedades analgésicas, antipiréticas y antiinflamatorias ^2^^,^^3^.

El uso correcto de antibióticos y antinflamatorios por parte de pacientes y profesionales de la salud es un pilar para reducir el desarrollo de resistencias y tolerancias, respectivamente. Según la OMS, el aumento en el uso de antibióticos es proporcional al número de cepas resistentes. En países como España, Francia y Portugal las cepas son más resistentes. Por ejemplo, en España, la concentración de neumococos resistentes a la penicilina es del 40% ^3^.

La prescripción de medicamentos en odontología pediátrica es uno de los puntos importantes en la práctica de esta especialidad, por lo que el profesional debe estar capacitado en el uso y dosificación correcta de los fármacos para evitar la administración innecesaria de medicamentos; para ello, debe tener conocimientos suficientes en farmacología y terapéutica. En este sentido, las evidencias internacionales sobre el tema señalan que los odontólogos no tienen una óptima preparación en farmacología ^4^^,^^5^.

El uso inadecuado e irracional de medicamentos en odontopediatría es uno de los factores más destacados en el desarrollo de la resistencia y la tolerancia a largo plazo de antibióticos antiinflamatorios y analgésicos. Por ese motivo, la OMS, en 1998, introdujo el término Terapéutica Razonada, así como el de Medicina Basada en la Evidencia (MBE), para mejorar las habilidades al momento de prescribir y tener mejores conocimientos en cuanto a farmacología. Actualmente, se han propuesto nuevas estrategias educativas con el fin de brindar una mejor calidad en la enseñanza y aprendizaje, para contribuir a un mejor desempeño profesional ^5^.

No existe en la actualidad una base de datos fiable y bien elaborada (historia clínica) mediante la cual podamos conocer los datos mínimamente aceptables, como si el paciente ha presentado o presenta alergias, si refiere alguna enfermedad sistémica, si está tomando en la actualidad algún medicamento o sus antecedentes, por lo que no es posible contar con la información mínima necesaria para recetar un medicamento ^2^.

Todo esto es muy importante para que los futuros profesionales (alumnos de pregrado) establezcan sus fundamentos teóricos y clínicos en el uso de antiinflamatorios, analgésicos y antibióticos en odontopediatría. Brindar un enfoque educativo amplio y con bases sólidas beneficiará la calidad de vida y, por ende, los problemas de salud desde la infancia temprana ^2^^,^^4^.

Por las razones expuestas, este estudio buscó evaluar los conocimientos en cuanto a prescripción de analgésicos, antiinflamatorios y antibióticos en odontopediatría, según sexo y año de estudios, a los alumnos de pregrado de odontología del VIII, X y XII ciclos de la Escuela de Odontología de la Universidad Católica Santo Toribio de Mogrovejo (Chiclayo, Perú).

## MATERIALES Y MÉTODOS

El presente estudio fue aprobado por el Comité de Ética de la Universidad Católica Santo Toribio de Mogrovejo, con resolución N.° 178-2023-USAT-FMED. Su diseño fue de tipo prospectivo, transversal, descriptivo y observacional. La población estuvo constituida por 86 estudiantes de pregrado del VIII, X y XII ciclo de la Escuela de Odontología, pertenecientes al año de estudios 2023-II, que iniciaron en agosto del mismo año en la Universidad Católica Santo Toribio de Mogrovejo. Finalmente, participaron 84 estudiantes, cuya información fue obtenida del portal transparencia de universidad ^6^. La investigación se realizó por medio de un cuestionario validado por expertos: un especialista en farmacología, dos odontopediatras y un especialista en salud pública. El cuestionario estuvo constituido por 11 preguntas sobre AINE y 11 preguntas sobre el uso de antibacterianos en odontopediatría. con alternativas múltiples y respuesta única. Cada respuesta correcta sumó 1 punto y las respuestas incorrectas, 0 puntos. El resultado se obtuvo sumando todas las interrogantes correctas y se clasificó del siguiente modo: insuficiente (0-6) y suficiente ^7^^-^^11^. Estuvo validado por un juicio de expertos ^7^, que tuvo como resultado una confiablidad alta, correspondiente al 0,708 ^7^.

Se procedió a elaborar una base de datos con la información obtenida de las encuestas anónimas que cumplieron con los criterios de inclusión y exclusión. Las variables que se tomaron en cuenta fueron el conocimiento sobre prescripción de antibióticos, antiinflamatorios y analgésicos, sexo y año de estudios. Finalmente, los datos se recopilaron en una base de datos de Excel 2019 y se analizaron en función de los objetivos de este estudio. El procesamiento de datos se realizó en una computadora con el siguiente software: Windows 11, Microsoft Word 2019, Microsoft Excel 2019 y el programa estadístico SPSS 25.

## RESULTADOS 

En la Tabla 1 se muestra la evaluación del nivel de conocimiento sobre analgésicos y antiinflamatorios en los estudiantes de pregrado, y se obtuvo como resultado un conocimiento insuficiente del 90,5% en el XII ciclo, seguido por el 82,9% de conocimiento insuficiente en el X ciclo y, finalmente, el 82,1% de conocimiento insuficiente en el VIII ciclo. Para el grupo de los antibióticos, resaltó el nivel de conocimiento insuficiente, con un porcentaje del 85,7% en el VIII ciclo, el 68,6% en el X ciclo y el 61,9% en el XII ciclo. 

**Tabla 1:** Nivel de conocimiento en estudiantes de pregrado del VIII, X, XII ciclo de odontología sobre prescripción de analgésicos, antiinflamatorios y antibióticos en odontopediatría, en una universidad privada de Chiclayo, 2023

Nivel de conocimiento							
		Insuficiente		Suficiente		Total	
		n	%	n	%	n	%
VIII ciclo	Analgésicos y antiinflamatorios	23	82,1	5	17,9	28	100,0
	Antibióticos	24	85,7	4	14,3	28	100,0
X ciclo	Analgésicos y antiinflamatorios	29	82,9	6	17,1	35	100,0
	Antibióticos	24	68,6	11	31,4	35	100,0
XII ciclo	Analgésicos y antiinflamatorios	19	90,5	2	9,5	21	100,0
	Antibióticos	13	61,9	8	38,1	21	100,0

En la Tabla 2 se presenta el nivel de conocimiento de los estudiantes de pregrado en cuanto a los analgésicos y antiinflamatorios, y se evidencia que, en el VIII ciclo, el nivel fue insuficiente para el 85,0% de estudiantes de sexo femenino y el 75,0% de estudiantes de sexo masculino. Asimismo, el nivel insuficiente de conocimientos para antibióticos fue del 85% para el sexo femenino y el 87,5% para el sexo masculino.

**Tabla 2:** Nivel de conocimiento en estudiantes de odontología del VIII, X, XII ciclo en cuanto a prescripción de analgésicos, antiinflamatorios y antibióticos en odontopediatría, en una universidad privada de Chiclayo, 2023, según sexo

Sexo				
			Femenino		Masculino		Total
			n	%	n	%	n
VIII ciclo	Analgésicos y antiinflamatorios	Insuficiente	17	85,0%	6	75,0%	23
		Suficiente	3	15,0%	2	25,0%	5
	Antibióticos	Insuficiente	17	85,0%	7	87,5%	24
		Suficiente	3	15,0%	1	12,5%	4
X ciclo	Analgésicos y antiinflamatorios	Insuficiente	23	85,2%	6	75,0%	29
		Suficiente	4	14,8%	2	25,0%	6
	Antibióticos	Insuficiente	18	66,7%	6	75,0%	24
		Suficiente	9	33,3%	2	25,0%	11
XII ciclo	Analgésicos y antiinflamatorios	Insuficiente	12	92,3%	7	87,5%	19
		Suficiente	1	7,7%	1	12,5%	2
	Antibióticos	Insuficiente	9	69,2%	4	50,0%	13
		Suficiente	4	30,8%	4	50,0%	8

Con relación a los analgésicos y antiinflamatorios en el ciclo X, el porcentaje de conocimiento insuficiente fue del 85,2% para el sexo femenino y el 75% para el sexo masculino. El nivel insuficiente de conocimientos para antibióticos fue del 66,7% para el sexo femenino y el 75% para el sexo masculino. Finalmente, respecto de los analgésicos y antiinflamatorios en el ciclo XII, el porcentaje de conocimiento insuficiente fue del 92,3% para el sexo femenino y el 87,5% para el sexo masculino, mientras que el nivel insuficiente de conocimientos para antibióticos fue del 69,2% para el sexo femenino y el 50% para el sexo masculino.

En la Tabla 3 se observa que el nivel de conocimiento sobre analgésicos y antiinflamatorios en estudiantes de pregrado alcanzó el mayor porcentaje (90,5%) en el XII ciclo (nivel de conocimiento insuficiente), seguido por el 82,9% en el X ciclo para analgésicos y antiinflamatorios (nivel de conocimiento insuficiente) y, finalmente, el 82,1% en el VIII ciclo (nivel de conocimiento insuficiente). 

**Tabla 3:** Nivel de conocimiento en estudiantes de pregrado del VIII, X, XII ciclo de odontología sobre prescripción de analgésicos, antiinflamatorios y antibióticos en odontopediatría, en una universidad privada de Chiclayo, 2023, según año de estudios

Año de estudios								
			VIII ciclo		X ciclo		XII ciclo	
			n	%	n	%	n	%
Nivel de conocimiento	Analgésicos y antiinflamatorios	Insuficiente	23	82,1	29	82,9	19	90,5
		Suficiente	5	17,9	6	17,1	2	9,5
	Antibióticos	Insuficiente	24	85,7	24	68,6	13	61,9
		Suficiente	4	14,3	11	31,4	8	38,1
Total			28	100,0	35	100,0	21	100,0

Asimismo, en el caso de los antibióticos, se obtuvo un mayor porcentaje (85,7%) en el VIII ciclo (nivel de conocimiento insuficiente), seguido por el 68,6% en el X ciclo (nivel de conocimiento insuficiente) y, por último, el 61,9% en el XII ciclo (nivel de conocimiento insuficiente).

## DISCUSIÓN

La población de esta investigación estuvo compuesta por estudiantes de pregrado de los ciclos VIII, X y XII de la escuela de Odontología de la Universidad Católica Santo Toribio de Mogrovejo (Chiclayo, Perú). El estudio tuvo como objetivo identificar el nivel de conocimientos sobre la prescripción de analgésicos, antiinflamatorios no esteroideos (AINE) y antibióticos en odontopediatría. Los resultados fueron los siguientes: para los medicamentos antiinflamatorios no esteroideos (AINE), un conocimiento insuficiente del 90,5% y suficiente del 9%; para los antibióticos, un grado de conocimiento insuficiente del 63,4% y suficiente del 36,6%. Se evidencia que el nivel de conocimiento predominante en ambos tipos de fármacos fue insuficiente. 

Con referencia al grado de conocimiento de los estudiantes de pregrado sobre los analgésicos y antiinflamatorios, se obtuvo un porcentaje de conocimiento insuficiente del 90,5% en el XII ciclo, seguido por un 82,9% en el X ciclo y un 82,1% en el VIII ciclo. En el caso de los antibióticos, el porcentaje de conocimiento insuficiente fue del 85,7% en el VIII ciclo, el 68,6% en el X ciclo y el 61,9% en el XII ciclo. Estos resultados discrepan de los encontrados por Fariña ^1^, quien evaluó alumnos del 5.o año que obtuvieron un 89% de nivel bueno en cuanto a conocimientos y un 11% de nivel regular. 

Con referencia a los niveles de conocimiento sobre los antibióticos, se obtuvo un porcentaje de conocimiento insuficiente del 85,7% en el VIII ciclo, del 68,6% en el X ciclo y del 61,9% en el XII ciclo. Esto se diferencia de lo obtenido por Altamirano ^2^, quien encontró un 96,70% para el nivel de conocimiento malo y un 3,3% para nivel de conocimiento regular. Estas diferencias se deben a los diversos niveles de conocimiento encontrados reflejados en las diversas metodologías de enseñanza de las facultades de odontología del país. 

En nuestro estudio, se observó un grado insuficiente en cuanto prescripción de antiinflamatorios, analgésicos y antibióticos en odontopediatría, lo que no concuerda por lo encontrado por Colque ^7^, quien halló que el grado de conocimiento para prescripción fue regular para antibióticos. 

Respecto de los porcentajes obtenidos sobre analgésicos, se halló un grado de conocimiento insuficiente en estudiantes de pregrado del 90,5% en el XII ciclo, el 82,9% en el X ciclo y el 82,1% en el VIII ciclo, lo que se diferencia de lo obtenido por Carhuancho ^8^, quien señala, para los alumnos del cuarto y quinto año evaluados, un grado insuficiente de conocimiento para prescripción de AINE en odontopediatría del 70,6% y el 82,5%, respectivamente. Esto se diferencia de los resultados en el sexto año, pues el grado predominante en cuanto a conocimiento en los alumnos fue el suficiente, con un 55,9%. Estos resultados difieren entre sí debido a que los estudiantes de nuestro estudio demuestran menos conocimientos sobre el tema en contraste con otros resultados encontrados. 

En cuanto a los porcentajes obtenidos sobre antibióticos en nuestro estudio, el nivel de conocimiento insuficiente se presentó en un 85,7% en el VIII ciclo, un 68,6% en el X ciclo y un 61,9% en el XII ciclo. Estos resultados se diferencian de lo encontrado por Carhuancho ^8^, quien observó que en los alumnos de 4.o y 5.o año predomina el nivel de conocimiento insuficiente sobre prescripción de antibióticos en odontopediatría, con el 68,6% y el 77,2%, respectivamente, mientras que en los estudiantes de 6.o año el nivel de conocimiento suficiente fue predominante, con un 67,6%. Estos resultados contrastan entre sí debido a que la variedad de conocimiento no es reforzada por la revisión de literatura, charlas, conferencias, boletines ni cursos de especialización que incrementen el conocimiento adquirido.

Sanga ^9^, al analizar el conocimiento sobre prescripción racional de antimicrobianos y analgésicos de los cirujanos dentistas de la ciudad de Juliaca, en 2018, obtuvo como resultado un nivel de conocimiento regular. Este mismo resultado se encontró en el estudio de Pérez ^10^ luego del análisis del nivel de conocimiento de los cirujanos dentistas sobre analgésicos, lo cual difiere de lo encontrado en nuestra población, que muestra un nivel de conocimiento deficiente, si bien se debe considerar que la población de estudio no cuenta con un grado académico, en comparación con la población considerada por Pérez, que ya tenía un grado académico.

Según De Vries *et al*. ^11^, la guía de la OMS indica el uso racional recomendado de analgésicos y antiinflamatorios para evitar la creación de resistencia y tolerancia a estos medicamentos. Esto permitiría reforzar el nivel deficiente encontrado en nuestro estudio. Ruiz ^12^, en su estudio, menciona que se debería tener una estrategia educativa que permita afianzar el manejo ambulatorio de las infecciones orales. Según los datos obtenidos en nuestro estudio, se debería evaluar la estrategia educativa para que los alumnos puedan mejorar su nivel de conocimiento.

Lifshitz ^13^ indica que la automedicación y la autoprescripción son prácticas inapropiadas a las que se atribuye consecuencias nocivas. En nuestro estudio, observamos que el nivel de conocimiento insuficiente podría ser perjudicial en el manejo de los medicamentos indicados. 

Bernabé ^14^ muestra que el nivel de conocimiento en cuanto a prescripción de antibióticos de su población tuvo carencias. Por su parte, Lauber ^15^ señala que los dentistas y los médicos muestran distintos niveles de comprensión en cuanto a prescripción para profilaxis antibiótica. Ambos estudios muestran carencias en cuanto a prescripción, lo mismo que se obtuvo en nuestro estudio. En otro sentido, Giunta ^16^ sugiere la contratación de odontopediatras en los hospitales para obtener un mejor abordaje en las infecciones de tipo odontogénico en los niños, lo que podría ser una desventaja si se mantienen los resultados encontrados en nuestro estudio. También debemos considerar lo que Amez ^17^ menciona respecto de que debemos mantener un manejo de dolor adecuado, así como un control adecuado de las enfermedades sistémicas. Acosta ^18^ manifiesta que, para lograr este buen manejo, se requiere una capacitación constate por parte del odontopediatra. De acuerdo con los resultados de la mayoría de los estudios, el manejo de la salud dental en pacientes con compromisos médicos es pobre, lo que puede ser tomado en consideración para desarrollar una preparación a nuestra población de estudio. 

También debemos considerar lo que Cortés ^19^ señala acerca de la necesidad de contar con un protocolo adecuado en cuanto a pacientes con cardiopatías, para lo cual considera una prescripción adecuada. Lo que debemos tener en cuenta es el nivel de conocimiento que se tiene, que en nuestra población de estudio fue insuficiente, y debería ser reforzado para abordar este tipo de patologías.

Al realizar una evaluación entre estudiantes de medicina y odontología, Shahroom ^20^ encontró que el nivel de conocimiento sobre prescripción de medicamentos entre los estudiantes de odontología y medicina es moderado, considerando que un 36% del total fueron estudiantes de odontología, quienes obtuvieron un nivel moderado. Esto contrasta con lo obtenido en nuestro estudio, donde se observa un nivel de conocimiento deficiente del 85%. Los resultados difieren por el tipo de variables que se consideraron en los diferentes estudios.

## CONCLUSIONES 

Los estudiantes de pregrado evaluados presentaron un nivel de conocimiento mayoritariamente insuficiente en el uso de analgésicos y antiinflamatorios, por lo que se sugiere implementar estrategias para reforzar estos conocimientos en el pregrado, a fin de mejorar su práctica. 

Cabe señalar que resultados obtenidos no se pueden generalizar, debido a que la población de estudio correspondió a una institución y ciclos específicos, y se debe considerar que cada universidad maneja una malla curricular y un enfoque educativo distintos.

## Fuentes de financiamiento:

Ninguna
